# Correlative light and electron microscopy of wall formation in *Eimeria nieschulzi*

**DOI:** 10.1007/s00436-020-06765-6

**Published:** 2020-07-06

**Authors:** Stefanie Wiedmer, Thomas Kurth, Ulrike Buder, Sinja Bleischwitz, Rolf Entzeroth, Michael Kurth

**Affiliations:** 1 Faculty of Biology, Institute of Zoology, Technische Universität Dresden, Zellescher Weg 20 B, 01217 Dresden, Germany; 2 Center for Molecular and Cellular Bioengineering (CMCB), Technology Platform, Technische Universität Dresden, Fetscherstraße 105, 01307 Dresden, Germany

**Keywords:** *Eimeria*, Gametocytes, Wall-forming bodies, Oocyst wall, GAM proteins, Correlative light and electron microscopy

## Abstract

Coccidian parasites possess complex life cycles involving asexual proliferation followed by sexual development leading to the production of oocysts. Coccidian oocysts are persistent stages which are secreted by the feces and transmitted from host to host guaranteeing life cycle progression and disease transmission. The robust bilayered oocyst wall is formed from the contents of two organelles, the wall-forming bodies type I and II (WFBI, WFBII), located exclusively in the macrogametocyte. *Eimeria nieschulzi* has been used as a model parasite to study and follow gametocyte and oocyst development. In this study, the gametocyte and oocyst wall formation of *E. nieschulzi* was analyzed by electron microscopy and immuno-histology. A monoclonal antibody raised against the macrogametocytes of *E. nieschulzi* identified a tyrosine-rich glycoprotein (EnGAM82) located in WFBII. Correlative light and electron microscopy was used to examine the vesicle-specific localization and spatial distribution of GAM82-proteins during macrogametocyte maturation by this monoclonal antibody. In early and mid-stages, the GAM82-protein is ubiquitously distributed in WFBII. Few hours later, the protein is arranged in subvesicular structures. It was possible to show that the substructure of WFBII and the spatial distribution of GAM82-proteins probably represent pre-synthesized cross-linked materials prior to the inner oocyst wall formation. Dityrosine-cross-linked gametocyte proteins can also be confirmed and visualized by fluorescence microscopy (UV light, autofluorescence of WFBII).

**Electronic supplementary material:**

The online version of this article (10.1007/s00436-020-06765-6) contains supplementary material, which is available to authorized users.

## Introduction

Coccidian parasites, e.g., *Toxoplasma gondii*, *Sarcocystis* spp., and *Eimeria* spp., are obligate intracellular parasites and pathogens of medical and economic importance. Coccidian oocysts are crucial for the survival of the parasites in the external environment and the transmission to suitable hosts (Kheysin 1972). The oocyst wall, which is formed from proteins synthesized during the macrogametocyte development, has unique characteristics that protect the enclosed sporozoites from chemical and physical damage (Kheysin 1972; Scholtyseck and Voigt 1964). Hence, oocysts are resistant to disinfectants and chemicals, like sulfuric acid or potassium dichromate (Dubey et al. 1970; Kheysin 1972; Marquardt 1966), although they are sensitive to heat, cold, and desiccation (Dubey 1998; Kheysin 1972; Ryley 1973).

Due to the properties of the oocyst wall, studies of oocyst development and wall formation are proving difficult. Therefore, the structure and formation of the oocyst wall are not yet fully understood. So far, it is known that in *Eimeria* maturation, two types of wall-forming bodies (WFBI and WFBII) arise which produce the material for the prospective two layers of the oocyst wall (Scholtyseck and Voigt 1964; Scholtyseck et al. 1971). A third layer, a loose outer veil, enclosing the maturing macrogametocyte and developing oocyst, was formed from granules of a third type, the veil-forming bodies (VFB; Ferguson et al. 1975, 2003; Pittilo and Ball 1980). The oocyst wall formation of coccidian parasites involves a number of steps: WFB (I and II) are located and inter-mixed in the cytoplasm of the macrogametocyte. After fertilization by a microgametocyte, macrogametocytes are developed into zygotes and the wall formation is initiated. WFBI are transferred to the periphery of the macrogametocyte, disaggregated and fused together to form the outer layer of the oocyst wall. Shortly after this, WFBII are located in the rough endoplasmic reticulum, transferred to the periphery and fused together, forming the inner oocyst wall (Ferguson et al. 2003; Mai et al. 2009; Scholtyseck et al. 1971).

However, few antibodies to specific proteins associated with gametocyte maturation and oocyst wall formation have been described and characterized (Belli et al. 2003a, b; Ferguson et al. 2000; Fried et al. 1992; Karim et al. 1996; Laxer et al. 1987; Mouafo et al. 2002; Walker et al. 2015; Wallach et al. 1989, 1990). Tyrosine-rich gametocyte proteins (GAM56, GAM82) could be identified and localized to WFBII and the inner oocyst wall of *E. tenella* (Belli et al. 2009; Mouafo et al. 2002), *E. acervulina* (Belli et al. 2009), and *E. maxima* (Belli et al. 2002a, b, 2003a, b, 2009). GAM precursor proteins containing tyrosine-rich domains are proteolytically processed into smaller peptides prior to protein–tyrosine cross-linking and oocyst wall hardening (Belli et al. 2003a, b; Belli et al. 2006). Hence, dityrosine cross-linking and hardening of the oocyst wall lead to the characteristic blue UV autofluorescence (Belli et al. 2003a, 2006; Wiedmer et al. 2018).

In this study, *Eimeria nieschulzi*, a rat-specific parasite, has been used as a model organism to investigate macrogametogenesis and oocyst wall synthesis. The use of antibodies in conjunction with immuno-histology, electron microscopy (EM), and correlative light and electron microscopy (CLEM) allowed a detailed analysis of macrogametocyte development and oocyst wall formation. Especially, CLEM is an excellent method to analyze the distribution of proteins in the context of cellular morphology and ultra-structure (Müller-Reichert and Verkade 2012, 2014). In one variation, CLEM is performed with immuno-labeled ultra-thin sections. Here, fluorescence microscopy is used to identify regions-of-interest (ROI) with the fluorescence-labeled protein in an ultra-thin tissue section which is subsequently contrasted and imaged in the electron microscope (Fabig et al. 2012; Loussert et al. 2012). In this study, we have used a specific monoclonal antibody (mAb) raised against macrogametocytes of *E. nieschulzi* to determine the vesicular location of tyrosine-rich glycoproteins within maturing macrogametocytes and developing oocysts by immuno-histology and CLEM.

## Material and methods

### Parasites

Oocysts of *Eimeria nieschulzi* were routinely passaged in rats (*Rattus norvegicus*, Sprague Dawley® Rat, Crl:SD; Charles River Laboratories, Inc.), according to Jonscher et al. (2015). Gametocytes were accumulated and purified from infected rats as described previously (Wiedmer et al. 2017). Briefly, macrogametocytes of *E. nieschulzi* were isolated from infected small intestines 149 h post infection (h p.i.) and were then purified and concentrated by mechanical separation, filtration, and discontinuous percoll density-gradient centrifugation (modified after Mouafo et al. 2002; Pugatsch et al. 1989). Macrogametocytes were washed (phosphate buffered saline (PBS); 3×, 1500 rpm, 10 min) and stored at − 80 °C.

Oocysts of *E. falciformis* and *E. papillata* were passaged and harvested in mice (*Mus musculus*, BALB/cAnNCrl; Charles River Laboratories, Inc.) according to Wiedmer et al. (2011) and Ernst et al. (1971), respectively.

### Immunization and production of monoclonal antibodies

Pure macrogametocytes of *E. nieschulzi* (5 × 10^6^; see above) were disrupted and homogenized on a vortexer using glass beads (Ø 0*.*25–0.5 mm, Carl Roth GmbH+Co.KG). The gametocyte suspension was mixed with an equal volume of Freund’s adjuvant (complete; F 5881, Sigma-Aldrich®) and injected subcutaneously into 7-week-old BALB/c mice (*Mus musculus*, BALB/cAnNCrl; Charles River Laboratories, Inc.; 100 μl on each body side; 23 G needle) on day 0. Homogenized macrogametocytes (see above), emulsified in Freund’s adjuvant (200 μl) (incomplete; F 5506, Sigma-Aldrich®), were further injected on days 21 and 47 (100 μl on each body side). Blood was collected from the *tail vein* by puncture (24 G needle) at days 0, 33, and 50 in eppendorf tubes. Serum was harvested by clotting (30 min, room temperature (RT)) and centrifugation (13000 rpm, 10 min). The generation of hybridoma cells was carried out according to Köhler and Milstein (1975) by fusion of splenocytes of immunized BALB/c mice with immortal X63AG8.653 mouse myeloma cells on day 50. Briefly, spleens of immunized mice were rinsed with Dulbecco’s Modified Eagle Medium (DMEM; 41965-039, gibco®) and dissected into smaller pieces. Tissues were pressed through a metal filter (mesh size, 400 μm) and rinsed with DMEM. Freshly harvested splenocytes and myeloma cells (4:3 ratio) were pelleted by centrifugation and fused by addition of polyethylene glycol 1450 (PEG 1450) to the pellet (addition of 1 ml PEG 1450 over 1 min, mixed gently, addition of 1 ml PEG 1450 over 3 min, addition of 7 ml PEG 1450 over 3 min). Fused cells were centrifuged again and resuspended in selection medium (2% sodium hypoxanthine, aminopterin, and thymidine (HAT; H0262, Sigma-Aldrich®), 10% fetal bovine serum, 2% l-glutamine, 2% penicillin/streptomycin, 1% HEPES, and 1% sodium pyruvate in DMEM), and aliquoted into 96-well microtiter plates. After 10 days, the HAT medium was replaced with sodium hypoxanthine and thymidine medium (HT; H0137, Sigma-Aldrich®). Hybridoma cells were then maintained in DMEM (with 10% fetal bovine serum, 2% l-glutamine, 2% penicillin/streptomycin, 1% HEPES, and 1% sodium pyruvate). Cell culture supernatants of hybridoma cells were screened for antigen-specific antibodies by immunofluorescence assays and western blots against macrogametocytes of *E. nieschulzi* (see below). Positive hybridoma cells were cloned by limited dilution, according to Coller and Coller (1983). Isotyping was performed with the IsoStrip Mouse Monoclonal Antibody Isotyping Kit (11 493 027 001, Roche Diagnostics GmbH) according to the manufacturer’s instructions.

### Affinity chromatography

Hybridoma cell culture supernatants (see above) were centrifuged (1500 rpm, 10 min) and filtered (pore size, 0.45 μm). For binding, 100 ml hybridoma cell culture supernatant was mixed with 5.8 g sodium chloride (NaCl) and 1.6 ml 3 M Tris buffer (pH 8.8). Then the sample was mixed with 600 μl Pierce™ Protein G agarose (20 398; Thermo Fisher Scientific Inc.) and added to the column (1–5-ml column, Thermo Fisher Scientific Inc., polyethylene filter (pore size, 30 μm)) for 1 h at 4 °C. After binding, the column was treated with PBS to remove all non-specific components (2×). Specific, purified antibodies were eluted from Protein G resin by adding elution buffer (50 mM glycine, 150 mM NaCl in H_2_O ention (pH 3*.*0)) and 60 μl 3 M Tris buffer (pH 8*.*8). The antibody was then dialyzed against PBS overnight (dialysis tubing cellulose membrane, MWCO 14,000; D9277, Sigma-Aldrich®). The quantitative evaluation of the antibody concentration was carried out using a protein quantitation assay according to Bradford (Roti® Quant; Carl Roth GmbH+Co.KG) following the manufacturer’s instructions. These purified antibodies were used to probe gametocytes in various microscopy and biochemical techniques (see below).

### Immunofluorescence assay

For the detection of macrogametocyte-specific antibodies, macrogametocytes of *E. nieschulzi* (149 h p.i.; see above), *E. falciformis* (166 h p.i.), and *E. papillata* (120 h p.i.) were air-dried on slides (diagnostic microscopic slides, ER-208B-AD-CE24, Thermo Fisher Scientific Inc.) overnight and permeabilized in acetone (− 20 °C, 20 min). The immunofluorescence assay was carried out according to Bauer et al. (1995). Briefly, air-dried macrogametocytes were blocked in 5% bovine serum albumin (BSA) (RT, 20 min), washed (PBS), and then incubated with undiluted cell culture supernatants (37 °C, 60 min). After washing (0.05% Tween20 in PBS; RT, 4 × 5 min), macrogametocytes were probed with an appropriate secondary antibody (Anti-Mouse Polyvalent Immunoglobulins (G,A,M)-FITC; F1010, Sigma-Aldrich**®**; 1:50 in PBS or Anti-Mouse IgG (whole molecule)-FITC; F0257, Sigma-Aldrich®; 1:50 in PBS; 37 °C; 60 min), washed, mounted with Mowiol (Carl Roth GmbH+Co.KG), and examined by fluorescence microscopy (Zeiss Axiovert 100 M microscope, Colibri.2-light source).

### SDS-PAGE and western blot

To analyze proteins from macrogametocytes of *E. nieschulzi*, intestinal epithelial cells, scrapings of infected or uninfected rat intestines, and heterologous expressed proteins were resuspended and heated in sample buffer (including mercaptoethanol, Schägger 2006; 95 °C, 15 min). After centrifugation (1500 rpm, 10 min), supernatants were used for sodium dodecyl sulfate-polyacrylamide gel electrophoresis (SDS-PAGE). Protein samples were separated by SDS-PAGE (Schägger and von Jagow 1987; Schägger 2006) and visualized by Coomassie Brilliant Blue–staining or blotted to nitrocellulose membrane by tank blotting (Towbin et al. 1979). The membranes were blocked (2.5% milk powder, 2.5% BSA in PBS; RT, 2 h) and then incubated with undiluted cell culture supernatants of hybridoma cells (RT, 2 h). After washing (3×, PBS), the proteins were subsequently probed with an appropriate secondary antibody (Anti-Mouse IgG (whole molecule)-peroxidase (A9044, Sigma-Aldrich®; 1:50,000 in PBS)) for 60 min at RT. Probed membranes were developed using the substrate 3,3′,5,5′-tetramethylbenzidine (SERVA Electrophoresis GmbH) according to manufacturer’s instructions.

### Tissue processing for light microscopy and electron microscopy

Intestine samples were washed with PBS and fixed with 4% paraformaldehyde in Sörensen phosphate buffer (pH 7.4; 4 °C, overnight) after dissection of the infected animals (120–194 h p.i) and dissected into smaller blocks before embedding. Samples were dehydrated in a graded series of ethanol/water, incubated and embedded either in epon (Embed 812), LR White resin (Wiedmer et al. 2018), Lowicryl (K4M) (Fabig et al. 2012), or paraffin (Chen et al. 2013). For embedding in epon, tissues were washed with Sörensen phosphate buffer, postfixed in 1% osmium tetroxide and washed again. Samples were dehydrated in ascending ethanol concentrations, transferred into propylene oxide, and infiltrated and embedded in epon.

To analyze the tissue area, semi-thin sections (300–800 nm) were prepared from epon, LR White resin, or Lowicryl (K4M)-embedded tissue (see above) (ultramicrotome Reichert Ultracut R, Reichert AG; glass knives) and stained with Richardson’s solution (Richardson et al. 1960) or toluidine blue/borax. Ultra-thin sections (70 nm) of these samples were prepared (ultramicrotome Leica, EM UC6; diamond knife (DiATOME)) and mounted on formvar-coated copper or nickel 200-mesh grids and stained with uranyl acetate (Lowicryl, LR White) or with lead citrate (Venable and Coggeshall 1965) and uranyl acetate (epon).

### Immuno-histology

Sections (5 μm) were produced from paraffin-embedded samples of *Eimeria*-infected animals (rotary microtome Leica RM2125RT). Deparaffinization of sections, antigen retrieval with sodium citrate buffer (10 mM; 0.05% Tween20; pH 6.0), and immunofluorescence assays were carried out according to Chen et al. (2013). The antibodies used were chosen based on the results of the immunofluorescence assay and were already listed above. Sections were examined by fluorescence microscopy (Zeiss Axiovert 100 M microscope, Colibri.2-light source). Image acquisition and processing were performed with an Olympus F-ViewII monochromatic CCD-camera and the CellF software (Olympus Europa SE & Co. KG).

### Recombinant protein expression

Genomic DNA was isolated from purified sporozoites of *E. nieschulzi* using the Nucleo-Spin® Tissue Kit (Macherey-Nagel GmbH & Co. KG), following the manufacturer’s instructions, purified with phenol chloroform extraction (Mülhardt 2009), and then used as a template for PCR. DNA sequences (*Engam*56_1, *Engam*56_2, *Engam*82) were amplified by PCR (primer sequences see Table 1) and cloned into the pLATE 52 plasmid (aLICator LIC cloning & expression system Kit 2; Nterminal His-tag/WQ; K1281, Thermo Fisher Scientific Inc.) according to the manufacturer’s recommendations and transformed in competent *E. coli* (DH10B) by heat shock (Mülhardt 2009). The heterologous expression constructs are listed in Table 2. Cloned sequences were confirmed by restriction analysis and sequencing (GATC Biotech AG; Konstanz, Germany). Recombinant protein expression from *E. coli* Rosetta DE3, grown in LBamp medium (100 μg/ml ampicillin) at 37 °C, was induced with 1 mM Isopropyl-β-d-thiogalactopyranosid (IPTG) at an absorbance OD_600_ between 0.5 and 0.6 (Thermo Fisher Scientific Inc.). Bacteria were incubated for 3 h, subsequently sedimented by centrifugation, and stored at − 80 °C.

**Table 1:** Primer sequences

No	Primer	Sequence
1.	p52EnGam56_1Nterm_FW	GGTTGGGAATTGCAAGAGCCCATCGGCGAGCCTGAAATCC
2.	p52EnGam56_1_RV	GGAGATGGGAAGTCATTATTTAGGACCCCAGGTGTATACACC
3.	p52EnGam56_1Nterm_RV	GGAGATGGGAAGTCATTACCTTTTTCCCATGTTCCGCATC
4.	p52EnGam56_2Nterm_FW	GGTTGGGAATTGCAAGAGCCTAGTACAGTTGAACGCG
5.	p52EnGam56_2Nterm_RV	GGAGATGGGAAGTCATTACCTCTTTCCCATGTTCCGCATC
6.	p52EnGam82_FW	GGTTGGGAATTGCAACTGCCCACTCTGGAAAATGC
7.	p52EnGam82_RV	GGAGATGGGAAGTCATTAGTTGTAGGTCGTTTCCCAGG
8.	p52EnGam82Nterm_RV	GGAGATGGGAAGTCATTACCTCTTACCCAGGTTGCGTTC
9.	p52EnGam82Cterm_FW	GGTTGGGAATTGCAAGAACGCAACCTGGGTAAGAGG
10.	p52EnGam82CN_RV	GGAGATGGGAAGTCATTACAAAGAGCGAGGAGCTGCGTTG
11.	p52EnGam82CC_FW	GGTTGGGAATTGCAATACGCAAGCTTCGCTCGTGG

**Table 2:** Heterologous expression constructs (*Engam*56_1, *Engam*56_2, *Engam*82)

No.	Expression construct	Primer no.	Amino acids
Construct 1	p52*Engam*56_1_Full	1/2	21–465
Construct 2	p52*Engam*56_1_N	1/3	21–257
Construct 3	p52*Engam*56_2_N	4/5	21–243
Construct 4	p52*Engam*82_Full	6/7	20–540
Construct 5	p52*Engam*82_N	6/8	20–237
Construct 6	p52*Engam*82_C	9/7	231–540
Construct 7	p52*Engam*82_CC	11/7	377–540
Construct 8	p52*Engam*82_CN	9/10	231–376

### Correlative light and electron microscopy

For correlative immuno-labeling, ultra-thin sections of Lowicryl (K4M)-embedded tissues were stained simultaneously with fluorescent and gold markers as described previously (Fabig et al. 2012).

Briefly, the macrogametocyte-specific antibody (A1G8, from mouse) was detected first with a rabbit-Anti-Mouse bridging antibody followed by protein A gold (10 nm). After postfixation with 1% glutaraldehyde, a secondary goat-Anti-Rabbit antibody conjugated with Alexa Fluor 488 was applied. Finally, DAPI was used to detect the nuclei. Grids were mounted in 50% glycerol/water, and fluorescence microscopy was performed with a Keyence Biozero 8000 microscope. Grids were demounted, washed several times in water, and contrasted with 4% uranyl acetate and dried before EM-inspection with a FEI Morgagni 268 (Thermo Fisher Scientific Inc.) or a Jeol JEM-1400Plus transmission electron microscope running at 80 kV.

## Results

### Macrogametocyte development and oocyst wall formation visualized by EM

**Fig. 1:**
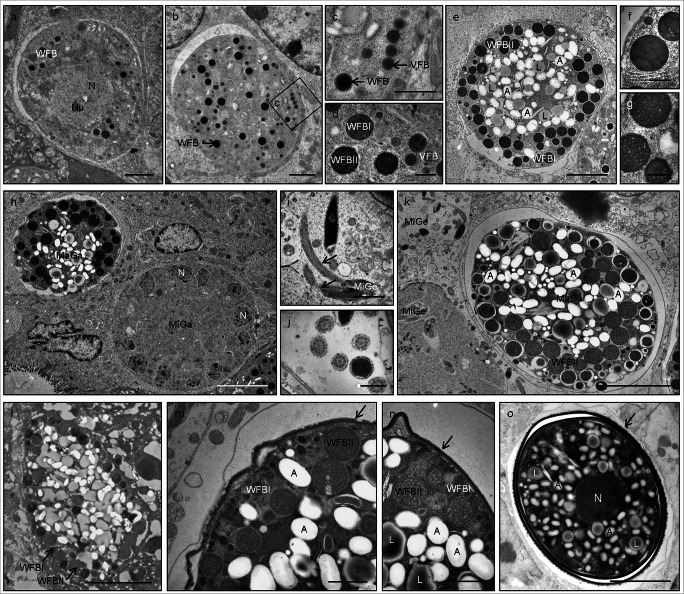
Transmission electron microscopy (TEM) images of developing and mature macrogametocytes, microgametocytes, and oocysts of *E. nieschulzi* in situ (149–159 h p.i) (**a**–**k**, **m**–**o** epon embedding, **l** LR White embedding). **a** Young macrogametocytes possess a large nucleus; wall-forming bodies (WFB) and polysaccharide granules are developed and located concentrically around the nucleus (149 h p.i.). **b** Young macrogametocyte, the cytoplasm contains immature homogeneous, osmiophilic, electron-dense WFB. **c** Higher magnification of (**b**). WFB and veil-forming bodies (VFB) are located in the peripheral cytoplasm of the parasite. **d**, **e** Mature macrogametocytes (158 h p.i.) show numerous large peripherally located WFBI, WFBII, and VFB. **f** The mature macrogametocyte contains osmiophilic WFBI, bound by a unit membrane. **g** WFBII consists of electron-dense filamentous materials. **h** Macrogametocytes (MaGa) and microgamonts (MiGa) are located in the epithelial cells in the small intestine of the rat. The microgamont (MiGa) contains numerous nuclei (N) (159 h p.i.). **i** Microgametocyte (MiGe) with flagella (arrow). **j** Longitudinal section of microgametocytes, each flagellum contains nine peripheral double microtubules and two central microtubules. **k**, **l** Mature macrogametocytes with peripherally located large WFB (159 h p.i.). WFBI, in different sizes, are inter-mixed with large WFBII. **m**, **n** Macrogametocytes show the developing outer and inner oocyst wall (arrow). The cytoplasm contains numerous large WFBII, polysaccharide granules and few small WFBI. Note the partially developed oocyst wall in (**m**) and the fully formed oocyst wall in (**n**) (arrow). **o** Fully developed oocyst, which is surrounded by a veil (arrow). A, amylopectin (polysaccharide granules); L, lipid droplets; MaGa, macrogametocyte (macrogamont); MiGa, microgamont; MiGe, microgametocyte; N, nucleus; Nu, nucleolus; VFB, veil-forming bodies; WFB, wall-forming bodies; WFBI, wall-forming bodies I; WFBII, wall-forming bodies II. Bar: (**a**, **b**, **e**) 2 μm, (**c**, **i**, **m**, **n**) 1 μm, (**d**, **f**, **g**, **j**) 500 nm, and (**h**, **k**, **l**, **o**) 5 μm

We investigated the ultra-structure and development of gametocytes and oocyst wall formation of *E. nieschulzi* in the rat small intestine. During the infection phase (149–159 h p.i.), young and mature macrogametocytes (Fig. 1a–h, k–n), microgamonts (Fig. 1h), microgametocytes (Fig. 1i–j) and unsporulated oocysts (Fig. 1o) can be observed in crypt epithelial cells. Young gametocytes are characterized by an even and round shape (Fig. 1a–b). In early stages of gametocyte development, macrogametocytes contain a centrally located nucleus, few polysaccharide granules, and numerous homogenous, osmiophilic vesicles which represent the early WFBII (Fig. 1a). Shortly after this, electron-dense granules appear in the peripheral cytoplasm; these organelles represent the early WFBI (Fig. 1b). In early and mid-stages, we found an additional type of membrane-bound vesicles called the veil-forming bodies (Fig. 1b–d). In matured macrogametocytes, these vesicles are secreted and form a veil on the outer surface of the maturing macrogametocyte and developing oocyst (Fig. 1m–o (arrow)). In mid-stages, WFBI are large, spherical, membrane-bound and osmiophilic structures, which are located in the periphery of the parasite (Fig. 1d–n) and contribute to the irregular shape of the parasite (Fig. 1h). WFBII are less electron dense than WFBI and have a loose, filamentous or sponge-like structure. They are arranged concentrically around the nucleus (Fig. 1d–n). Numerous polysaccharide granules and lipid droplets are also found in the cytoplasm of mature macrogametocytes (Fig. 1e, h, k). After fertilization by a microgametocyte (Fig. 1i–j), macrogametocytes develop into zygotes (Fig. 1m–o), and shortly after this, the oocyst wall starts to form (Fig. 1m–n (arrow)) with WFBI aligning under the pellicle of the zygote (Fig. 1l–n).

### Characterization of the EnGAM82 protein by immunofluorescence assays and SDS-PAGE

**Fig. 2:**
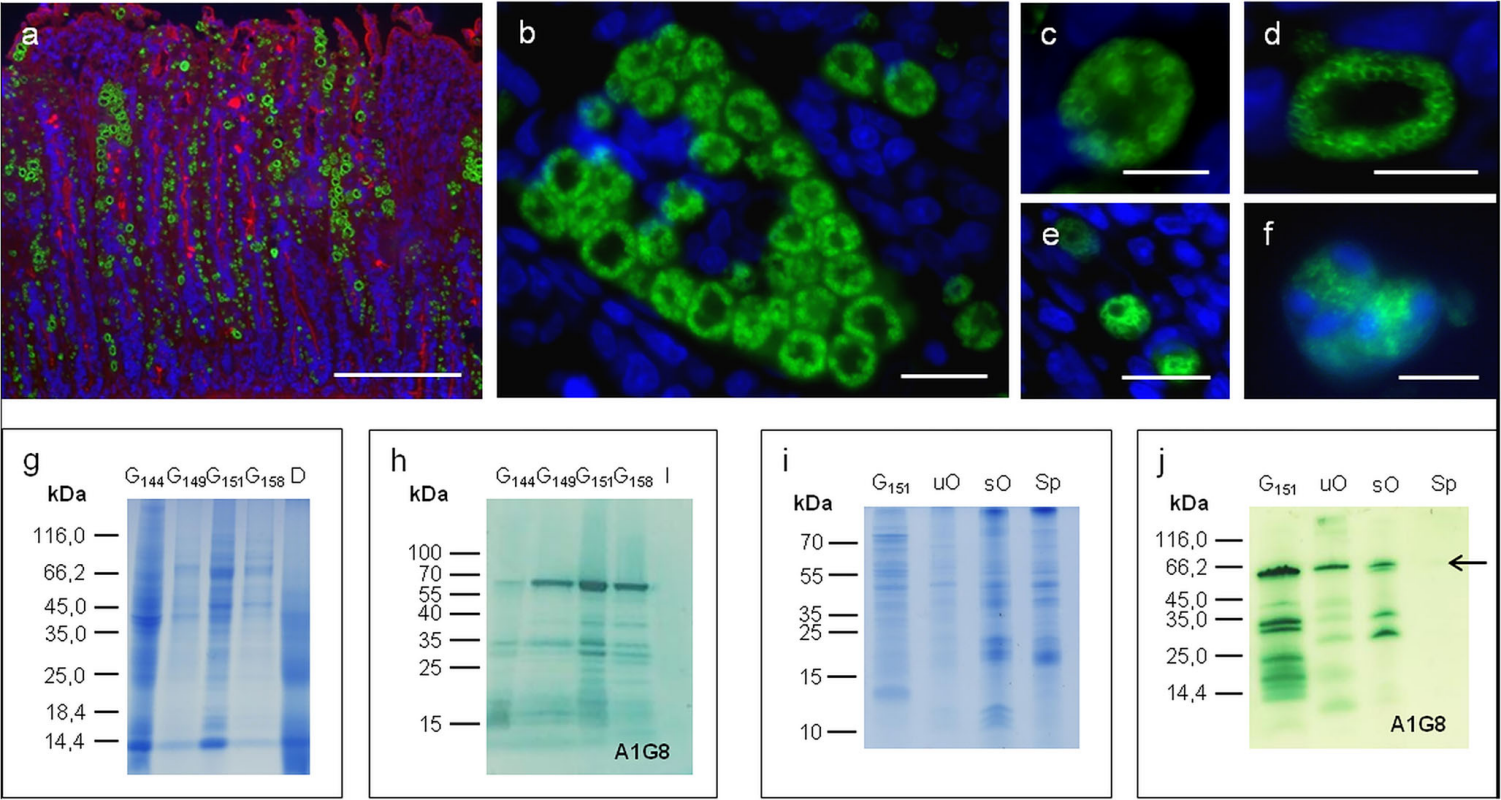
Paraffin-embedded sections of *E. nieschulzi* in situ (**a**–**e**), showing various developmental stages of macrogametocytes and developing oocysts as well as sporulated oocysts (**f**) (immuno-labeled with mAb A1G8, visualized with Anti-Mouse IgG-FITC and counterstained with DAPI) and analysis by SDS-PAGE and immuno-blotting (mAb A1G8) of macrogametocytes and oocysts (**g**–**j**). **a** Paraffin-embedded section showing various macrogametocytes at different developmental stages (159 h p.i.) containing punctiform or circle-shaped WFBII (green), counterstained with DAPI (nuclei) and WGA-FITC (goblet cells). **b**–**d** The circle-shaped WFBII are located in the periphery of the macrogametocyte (**b**, 148 h p.i.; **c**, 149 h p.i.; **d**, 194 h p.i.). **e** Young unsporulated oocysts with green fluorescent signals in the cytoplasm (194 h p.i.). **f** Sporulated oocyst, FITC signals are located in sporocysts. Bar: (**a**) 200 μm, (**b**) 20 μm, (**c**, **d**, **f**) 10 μm, (**e**) 40 μm. Analysis by SDS-PAGE (**g**, **i**) and immuno-blotting using the mAb A1G8 (**h**, **j**) revealed that the mAb recognized three polypeptides (30, 35, and 65 kDa) in young to mature macrogametocytes (144–158 h p.i.), various polypeptides in unsporulated (12–65 kDa) and sporulated oocysts (30, 35, 65 kDa). The antibody also detected a polypeptide of 65 kDa in sporozoites (arrow). G_144 gametocytes (144 h p.i.); G_149 gametocytes (149 h p.i.); G_151 gametocytes (151 h p.i.); G_158 gametocytes (158 h p.i.); I, uninfected epithelial cells of the small intestine (*Rattus norvegicus*, negative control); uO, unsporulated oocysts; sO, sporulated oocysts; Sp, sporozoites

In order to examine oocyst wall proteins of *E. nieschulzi*, gametocyte-specific antibodies were generated by hybridoma technology. One hybridoma cell–producing antibody directed against WFB of *E. nieschulzi* was then characterized. The mAb, named A1G8 (IgG1 isotype, antibody concentration: 79.62 μg/ml), reacted with vesicle-like structures in macrogametocytes (Fig. 2a–d), the cytoplasm of unsporulated oocysts (Fig. 2e), and appeared to be detecting the residual bodies in sporulated oocysts (Fig. 2f) by immunofluorescence assays. Analysis by SDS-PAGE (Fig. 2g, i) and western blot (Fig. 2h, j) revealed that the mAb recognized three distinct polypeptides at 30, 35, and 65 kDa in young and mature macrogametocytes as well as in unsporulated and sporulated oocysts (Fig. 2h, j). Staining with the mAb in uninfected rat intestinal cells revealed no bands in the western blot analysis (Fig. 2h (line I)). The antibody also detected a polypeptide of 65 kDa in sporozoites (Fig. 2j (line Sp)), which can be also observed by immunofluorescence assays (data not shown). Three candidate proteins (EnGAM56_1, EnGAM56_2, EnGAM82) emerged in that size (65 kDa; Fig. 2h, j), and therefore, three *gam*-genes (*Engam*56_1, *Engam*56_2, and *Engam*82; Wiedmer et al. 2017) were heterologous expressed as His-tagged proteins in *E. coli*. By immuno-blotting of bacterial cell lysates using the mAb A1G8, EnGAM82 was identified as the antibody binding site (epitope mapping) (EnGAM82-C-terminus; Figs. 5 and 6).

To investigate the cross-reactivity of this antibody with gametocyte proteins of two mouse species, immunofluorescence assays were used. WFB of *E. falciformis* and *E. papillata* were labeled with the mAb A1G8 in the same staining pattern (Fig. 7) to that observed in *E. nieschulzi* (Fig. 2).

### Oocyst wall synthesis visualized by LM and EM

**Fig. 3:**
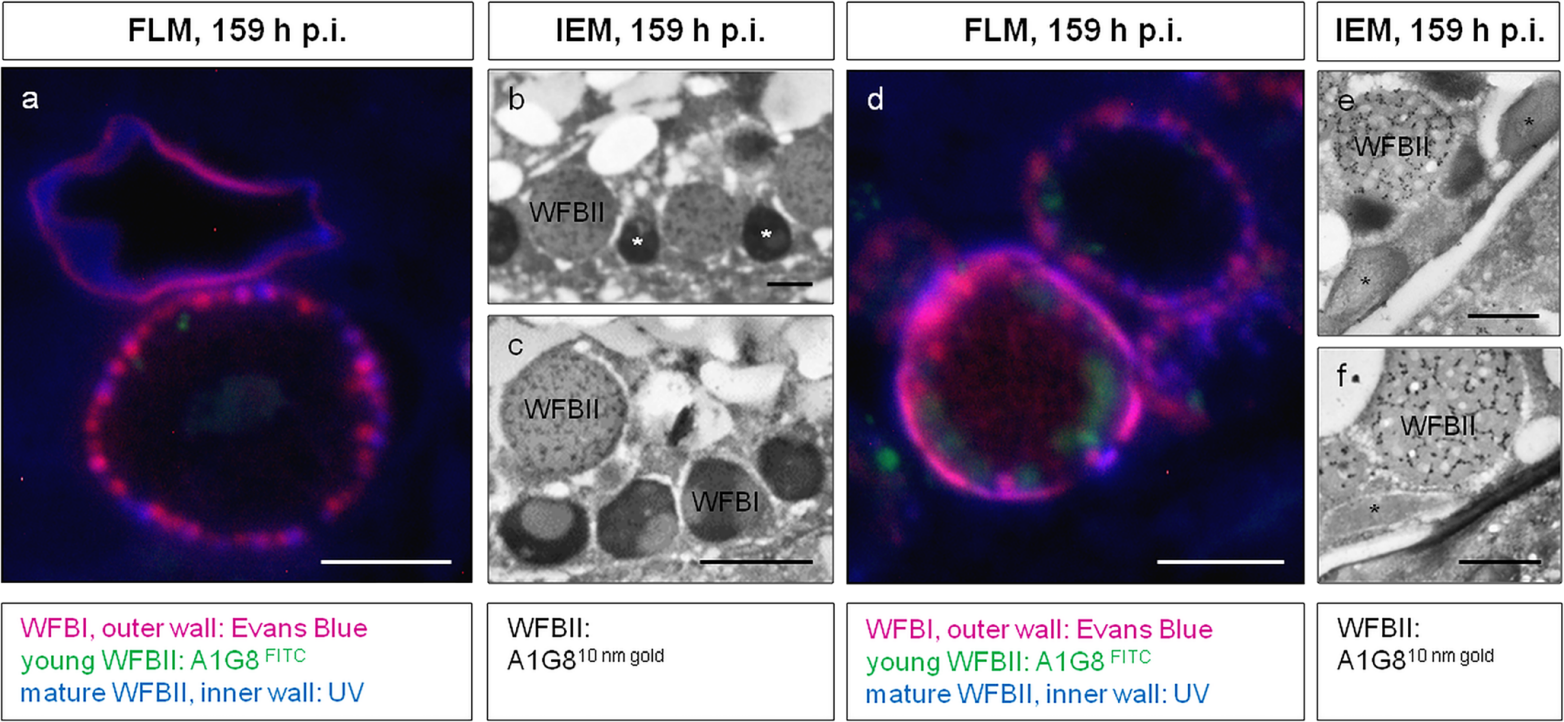
Fluorescence light microscopy (FLM) (**a**, **d**) and immuno-electron microscopy (IEM) (**b**–**c**, **e**–**f**) of *E. nieschulzi* in situ (159 h p.i.) (**a**, **d**, LR White embedding; **b**–**c**, **e**–**f**, Lowicryl embedding). **a**, **d** FLM, mature macrogametocytes and developing oocysts which have been stained with Evans Blue dye and immuno-labeled with the macrogametocyte-specific antibody A1G8. The Evans Blue dye appeared to stain WFBI and the outer layer of the oocyst wall (red), whereas young and mature WFBII can be labeled with mAb A1G8, visualized by Anti-Mouse IgG-FITC (green). Additionally, mature WFBII and the inner layer of the oocyst wall can be visualized by UV autofluorescence (UV light, blue). Mature macrogametocytes contain peripherally located large WFBI and WFBII (**a**, **d**). Macrogametocytes (**d**) show the partially formed outer and inner oocyst wall, probably the fusion of WFB to form the oocyst wall as a simultaneous process. The cytoplasm contains also numerous young WFBII (A1G8^FITC^, green). At this stage, the formation of the oocyst wall is still not completed. **b**–**c**, **e**–**f** IEM micrographs showing details of the periphery of mature macrogametocytes. WFBII are immuno-gold-labeled (A1G8^10nm gold^). WFBI (*) and WFBII are inter-mixed in the cytoplasm near the pellicle (**b**) or located among each other (**c**). **e** WFBI are flattened to initiate the fusion and formation of the outer oocyst wall, while the WFBII are unchanged in this region. **f** In one part of the macrogametocyte, the outer wall has been formed, while in the region next to it, the horizontally flattened WFBI are visible. WFBII are unchanged. WFBI, wall-forming bodies I; WFBII, wall-forming bodies II. Bar: (**a**, **d**) 10 μm, (**b**–**c**) 1 μm, (**e**–**f**) 500 nm

Immunocytochemistry on plastic sections was used to visualize the WFB and their spatial distribution in *E. nieschulzi* macrogametocytes. WFBI can be positively stained by Evans blue dye (Fig. 3a, d); WFBII can be detected with a macrogametocyte-specific antibody A1G8, and mature WFBII through their autofluorescence under UV-light (Fig. 3a, d; Wiedmer et al. 2018). In mature macrogametocytes, WFBI (red, Evans blue) and WFBII (blue, autofluorescence; green, mAb A1G8) are located either alternating in the periphery or forming two neighboring circles along the membrane (Fig. 3a, d). Simultaneously, WFBI and WFBII are inter-mixed in the periphery of the parasite (Fig. 3a, d) or located among each other (Fig. 3d) where they fuse to form the oocyst wall. This has been confirmed by immuno-electron microscopy of ultra-thin sections labeled with the WFBII-specific antibody A1G8 (Fig. 3b–c, e–f). The TEM-images also showed that, during this process, WFBI appeared to be horizontally flattened, and that they disaggregated and finally fused together to form an osmiophilic outer layer of the oocyst wall (Fig. 3e–f). Gaps in the outer oocyst wall are seen, while the inner wall appears to be completely formed (Figs. 1m (arrow), 3d). This process occurs in some sites earlier than in others, so that intact WFB are seen in areas next to those in which the vesicles have been flattened or fused (Figs. 1, 3). After the completion of this process, WFB are no longer present in the developing oocyst (Fig. 1o). The results of the EM study (Figs. 1, 3b–c, e–f) therefore confirm the spatial distribution of WFB in developing macrogametocytes and the simultaneous wall formation process visualized by fluorescence microscopy (Fig. 3a, d; Wiedmer et al. 2018).

### Spatial distribution of EnGAM82 proteins in WFBII visualized by CLEM

**Fig. 4:**
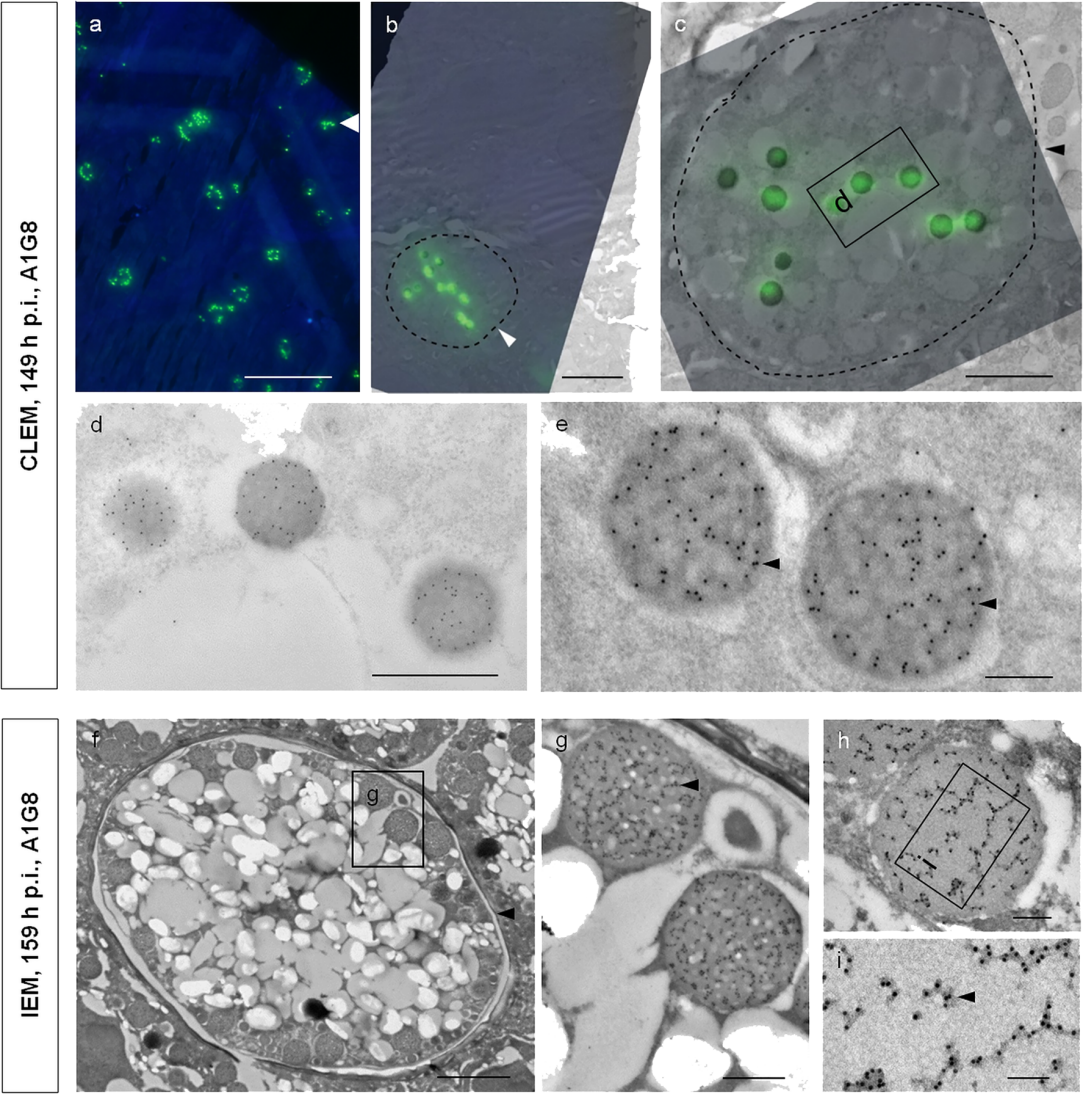
CLEM and IEM images of *E. nieschulzi* in situ using the mAb A1G8 visualized with Anti-Mouse IgG-FITC and Protein A gold (10 nm) (Lowicryl embedding). Electron microscopic images of ultra-thin sections of young and mature macrogametocytes (**a**–**e**, 149 h p.i.; **f**–**i**, 159 h p.i.). **a** FLM micrograph showing macrogametocytes, which have been immuno-stained with mAb A1G8 and visualized with FITC, counterstained with DAPI. **b** Overlay of the FLM and EM image of a macrogametocyte from (**a**), (arrow). **c** Higher magnification of the macrogametocyte from (**b**). Few small WFBII, located in the cytoplasm, are FITC labeled. **d** TEM micrograph of WFBII of the macrogametocyte from (**c**). **e** Detail from another macrogametocyte, WFBII are immuno-gold-labeled. The signals (EnGAM82 - proteins) are ubiquitously distributed in sponge-like structures of WFBII (arrow). **f**–**i** IEM of another macrogametocyte. **f** Mature macrogametocyte showing peripherally located WFBII with probably pre-synthesized proteins (EnGAM82). **g** Higher magnification of the WFBII from (**f**). **h**–**i** Part of a mature macrogametocyte, showing numerous gold particles in the WFBII. EnGAM82 proteins are arranged in subvesicular structures of the WFBII. Bar: (**a**) 50 μm, (**b**) 5 μm, (**c**) 2 μm, (**d**, **g**) 500 nm, (**e**, **h**) 200 nm, (**f**) 2.5 μm, and (**i**) 100 nm. FLM, Fluorescence light microscopy; IEM immuno-electron microscopy

Interestingly, Ferguson et al. (2003) reported differences in the distribution of tyrosine-rich glycoproteins in WFBII. These differences became apparent depending on the antibody used to detect them (Anti-GAM56, Anti-GAM82, Anti-GAM230, Anti-APGA (affinity-purified gametocyte antigen preparation); Ferguson et al. (2003)). CLEM of sections immuno-labeled with A1G8 was performed to study the vesicular localization of gametocyte-specific proteins in *E. nieschulzi* (Fig. 4). In this way, we performed direct correlative imaging (CLEM) of the same ultra-thin sections that were immuno-labeled with A1G8 as well as fluorescence- and gold-conjugated markers (Fig. 4a–c (fluorescence-conjugated marker), d–i (gold-conjugated marker)). Thus, the same subcellular structures (WFB within macrogametocytes) can be analyzed by fluorescence and electron microscopy. In early macrogametocytes (149 h p.i.), it was possible to show that the GAM82-protein is ubiquitously and homogenously distributed in WFBII (Fig. 4d, e). As the macrogametocyte matured (159 h p.i.), GAM82-proteins were arranged in subvesicular structures of WFBII (Figs. 3b–c, e–f, 4f–i). These signals were specific and could be attributed to the wedge or sponge-like structures of WFBII. No labeling was seen in WFBI, the cytoplasm, or polysaccharide granules (Fig. 4).

## Discussion

In this study, we used a WFB-specific antibody to label sections through a rat intestine infected with the coccidian parasite *E. nieschulzi*. By comparing the expression data with EM-images from tissue samples (Fig. 1–3), it could be shown that this semi-correlative approach allowed a comprehensive study of macrogametocyte maturation and oocyst wall formation of *E. nieschulzi*. To increase correlation and to validate our findings, we also performed direct correlative imaging of the very same ultra-thin sections that were immuno-labeled with WFB-specific antibodies as well as fluorescence- and gold-conjugated markers for CLEM (Fig. 4). In this way, the same subcellular structures can be analyzed by both light and electron microscopy. To our knowledge, this direct CLEM approach was used here for the first time to analyze the distribution of proteins necessary for oocyst wall formation in an *Eimeria* species.

The development and formation of the oocyst have been reported at the ultra-structural level in various coccidian parasites (Ferguson et al. 1975, 1979; Mehlhorn 1972; Pittilo and Ball 1980; Scholtyseck and Voigt 1964; Scholtyseck et al. 1966, 1969a, b, 1971). Macrogametocytes of most *Eimeria* species are relatively uniform in their ultra-structure (Scholtyseck et al. 1971) as well as *E. nieschulzi* macrogametocytes which will be presented here and confirm the results of further studies (Sibert 1978; Sibert and Speer 1980). However, there are also species-specific differences in relation to the number of membranes, the size and fine structure of WFB within macrogametocytes (Mehlhorn 1972; Scholtyseck et al. 1971; Sibert 1978; Sibert and Speer 1980), and the chronological formation of the oocyst wall (Ferguson et al. 2003; Wiedmer et al. 2018).

Tyrosine-rich glycoproteins like GAM56 and GAM82 are the best characterized proteins to date (Belli et al. 2006; Wallach et al. 1989). Their common feature is the characteristic amino acid tyrosine (Belli et al. 2006). Recent studies also describe a tyrosine-rich protein, EtSWP1, in the sporocyst wall of *E. tenella* (Walker et al. 2016) and tyrosine-rich proteins in *Toxoplasma gondii* (Fritz et al. 2012). Additionally, the GAM82 protein (EnGAM82) was recently described in *E. nieschulzi* (Wiedmer et al. 2017). Compared with avian GAM82 proteins, EnGAM82 also contains two tyrosine-rich domains surrounded by conserved motives (RxL, RRL, RRxG; Wiedmer et al. 2017). Noticeable differences are visible in their amino acid composition, which is not conserved over large regions (Wiedmer et al. 2017).

Previous studies investigating immunogenic properties of *E. maxima* macrogametocyte proteins in mice, rabbits, and chicken have shown that the proteins GAM56 and GAM82 are highly antigenic. Furthermore, the affinity-purified EmGAM56 and EmGAM82 antigens were used to immunize breeder hens, and it was reported that they stimulate the production of IgG antibodies (Pugatsch et al. 1989; Wallach et al. 1989, 1990, 1992, 1995). In this study, crude gametocyte extracts of *E. nieschulzi* (whole macrogametocytes) were used for immunization and antibody production in mice. It was shown by epitope mapping, that the antibodies, produced from immunized mice, are directed against EnGAM82; this suggests that it is also immunogenic in rodent coccidian parasites.

A previous study reported that gametocyte proteins, like GAM56 (EmGAM56) and GAM82 (EmGAM82) of *E. maxima*, with masses of 52.45 and 62.24 kDa, respectively, could not migrate to scale by SDS-PAGE due to its unusual amino acid composition (Belli et al. 2002a). Furthermore, the heterologously expressed and native GAM22 proteins of *E. necatrix* (EnGAM22) also failed to migrate to scale by SDS-PAGE (Liu et al. 2014). Similarly, in crude gametocyte extracts as well as in bacterial cell lysates expressing the EnGAM82 plasmid construct, a 65 kDa band was recognized in contrast to an expected size of 59.5 kDa. This might be also explained by an unusual amino acid composition, as in EmGAM82 and EnGAM22.

By immuno-blotting with A1G8 as a marker, three predominant bands were clearly identified, as well as various smaller proteins. This might be explained by the observation that these smaller proteins could be breakdown proteins or proteolytically processed peptides of EnGAM82 because they were recognized by the mAb A1G8. Thus, different protein patterns have been observed in young and mature gametocytes as well as in oocysts by immuno-blotting with mAb A1G8. Similar results have been found in avian parasites like *E. maxima* (Belli et al. 2009; Pote et al. 1991), *E. tenella* (Belli et al. 2009; Mouafo et al. 2002) and *E. acervulina* (Belli et al. 2009).

Immunofluorescence and immuno-electron microscopy studies showed that the fine structure of macrogametocytes and oocysts and the formation of the oocyst wall are strongly conserved across avian *Eimeria* species (*E. maxima*, *E. tenella*, and *E. acervulina*; Belli et al. 2009; Ferguson et al. 2003). Based on the sequence analysis, it was demonstrated that homologs of *E. maxima* macrogametocyte proteins (EmGAM56 and EmGAM82) are present in *E. tenella* and *E. acervulina* (Belli et al. 2009). Thus, antibodies directed against *E. maxima* proteins cross-react with WFB within macrogametocytes of all examined avian *Eimeria* species (Belli et al. 2009). In this study, the mAb A1G8 raised against the EnGAM82 proteins also cross-react with WFBII of other rodent *Eimeria* species (*E. papillata*, *E. falciformis*), as shown by immunofluorescence microscopy. This result can be explained by the fact that GAM56 and GAM82 proteins are closely related across rodent *Eimeria* species (*E. nieschulzi*, *E. falciformis*), as shown by sequence analysis (Wiedmer et al. 2017).

Ferguson et al. (2003) investigated macrogametocyte development and oocyst wall formation in *E. maxima* by immuno-histology and immuno-electron microscopy with antibodies raised against WFB-specific proteins. The authors used gametocyte-specific antibodies as tools to follow the synthesis, transport, and secretion of proteins during oocyst development (Ferguson et al. 2003). This study has shown that the oocyst wall formation requires a coordinated synthesis of the VFB and WFB in different cell compartments as well as a release of their contents probably controlled at the level of the rER and Golgi. Furthermore, Ferguson et al. (2003) showed substructures of WFB that probably represents differences in the protein distribution. The function of these substructures is still unknown. Thus, they postulated that the inner and outer oocyst wall formed from pre-synthesized materials of WFB (Ferguson et al. 2003). In this way, the spatial distribution of EnGAM82-proteins in WFBII confirms the findings of this earlier study (Ferguson et al. 2003). Additionally, dityrosine cross-linked proteins of coccidian parasites could be visualized by UV light (Belli et al. 2006; Daugschies et al. 2001; Frölich et al. 2013; Frölich and Wallach 2016; Wiedmer et al. 2018). Recently, a study focusing on the identification of a cysteine-rich protein (EnOWP13) of *E. nieschulzi* has shown that the autofluorescence is limited to the inner oocyst wall emerging from WFBII (Wiedmer et al. 2018). Based on this finding and the observation that pre-synthesized proteins (EnGAM82) were found in WFBII, we can assume that the dityrosine cross-linking starts prior oocyst wall formation and hardening. Furthermore, they also noted that the wall formation process of *E. nieschulzi* occurs simultaneously in contrast to avian *Eimeria* species (Wiedmer et al. 2018). Our electron microscopic observations confirmed these previous results.

In summary, by applying various microscopy techniques as immuno-histology, EM, and CLEM, it was possible to comprehensively show the formation of the oocyst wall of a rodent coccidian parasite. For the first time, a rodent macrogametocyte-specific antibody was generated. Thus, the newly generated antibody (Anti-EnGAM82; A1G8) was used as a specific, high-affinity marker to label WFBII-specific proteins. This is the only mAb so far which is directed against EnGAM82 and other orthologous proteins of rodent *Eimeria* species. Furthermore, the antibody-binding epitope (EnGAM82C) is well-known, and the cross-reactivity against other rodent *Eimeria* species is also be confirmed (*E. papillata*, *E. falciformis*).

## Electronic supplementary material

Fig. 5 Expression of recombinant EnGAM56_1, EnGAM56_2 and EnGAM82 peptides and epitope mapping using the monoclonal antibody A1G8 (a-b). Analysis by SDS-PAGE and immunoblotting revealed that the antibody A1G8 directed against the macrogametocytes of *E. nieschulzi* recognized the recombinant peptides of EnGAM82. **a 1** EnGAM56_2_N (−), **2** EnGAM56_2_N (+) (**construct 3**), **3** EnGAM56_1_Full (−), **4** EnGAM56_1_Full (+) (**construct 1**), **5** EnGAM56_1_N (−), **6** EnGAM56_1_N (+) (**construct 2**), **7** EnGAM82_Full (−), **8** EnGAM82_Full (+) (**construct 4**), **9** Rosetta DE3 (negative control), **10***E. nieschulzi* gametocytes 149 h p.i. **b 1** EnGAM82_Full (−), **2** EnGAM82_Full (+) (**construct 4**), **3** EnGAM82_N (−), **4** EnGAM82_N (+) (**construct 5**), **5** EnGAM82_C (−), **6** EnGAM82_C (+) (**construct 6**), **7** EnGAM82_CN (−), **8** EnGAM82_CN (+) (**construct 8**), **9** EnGAM82_CC (−), 10 EnGAM82_CC (+) (**construct 7**), 11 Rosetta DE3 (negative control) (PNG 1904 kb)

High-resolution image (TIF 4871 kb)

Fig. 6 Schematic illustration of heterologous expressed recombinant proteins: EnGAM56_1, EnGAM56_2, EnGAM82 and the truncated proteins of EnGAM82 (N- and C-terminus). By immunoblot of bacterial cell lysates using the mAb A1G8, the peptide EnGAM82CC (construct 7) was identified as the A1G8-epitope containing peptide (PNG 1641 kb)

High-resolution image (TIF 6565 kb)

Fig. 7 FLM images of paraffin-embedded sections of *E. papillata* (a-c) and *E. falciformis* (d-f) in situ, immuno-stained with mAb A1G8, visualized with Anti-Mouse IgG-FITC and counterstained with DAPI. a Macrogametocytes of *E. papillata* at different developmental stages containing punctiform or circle-shaped WFBII (green), counterstained with DAPI (nuclei). **b-c** Circle-shaped WFBII (green) are located in the cytoplasm of the macrogametocyte (small intestine, 120 h p.i.). **d-f** Macrogametocytes of *E. falciformis*, containing circle-shaped WFBII (green), are located in crypt epithelial cells of the colon (166 h p.i.). Bar: (a) 500 μm, (c) 20 μm, (d, e) 200 μm, (f) 20 μm. FLM fluorescence light microscopy (PNG 1220 kb)

High-resolution image (TIF 7613 kb)
