# Supplementary figures and images for: Correlative light and electron microscopy of wall formation in Eimeria nieschulzi
Source: Parasitol Res. 2020 Jul 6;119(8):2667–78. doi: 10.1007/s00436-020-06765-6 (PMC7366593; doi:10.1007/s00436-020-06765-6)

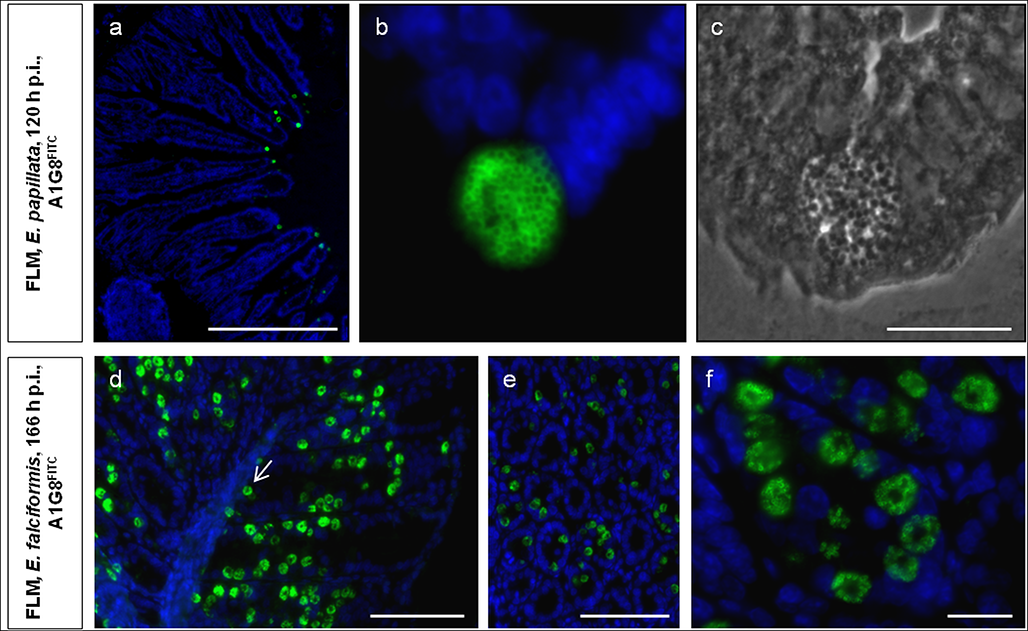

Supplement: Supplementary file 1 — Expression of recombinant EnGAM56_1, EnGAM56_2 and EnGAM82 peptides and epitope mapping using the monoclonal antibody A1G8 (a-b). Analysis by SDS-PAGE and immunoblotting revealed that the antibody A1G8 directed against the macrogametocytes of E. nieschulzi recognized the recombinant peptides of EnGAM82. a 1 EnGAM56_2_N (−), 2 EnGAM56_2_N (+) (construct 3), 3 EnGAM56_1_Full (−), 4 EnGAM56_1_Full (+) (construct 1), 5 EnGAM56_1_N (−), 6 EnGAM56_1_N (+) (construct 2), 7 EnGAM82_Full (−), 8 EnGAM82_Full (+) (construct 4), 9 Rosetta DE3 (negative control), 10E. nieschulzi gametocytes 149 h p.i. b 1 EnGAM82_Full (−), 2 EnGAM82_Full (+) (construct 4), 3 EnGAM82_N (−), 4 EnGAM82_N (+) (construct 5), 5 EnGAM82_C (−), 6 EnGAM82_C (+) (construct 6), 7 EnGAM82_CN (−), 8 EnGAM82_CN (+) (construct 8), 9 EnGAM82_CC (−), 10 EnGAM82_CC (+) (construct 7), 11 Rosetta DE3 (negative control) (PNG 1904 kb) [file 436_2020_6765_Fig5_ESM.png]

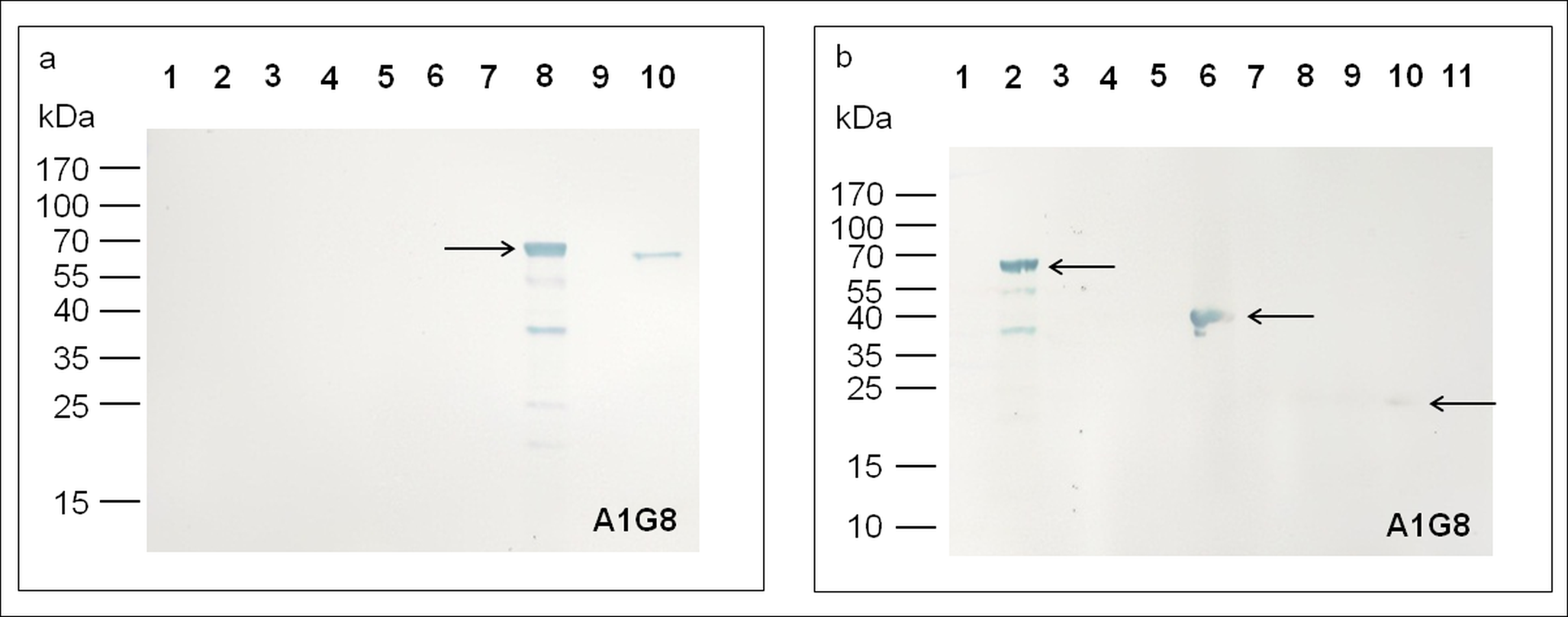

Supplement: Supplementary file 2 — High-resolution image (TIF 4871 kb) [file 436_2020_6765_MOESM1_ESM.tif]

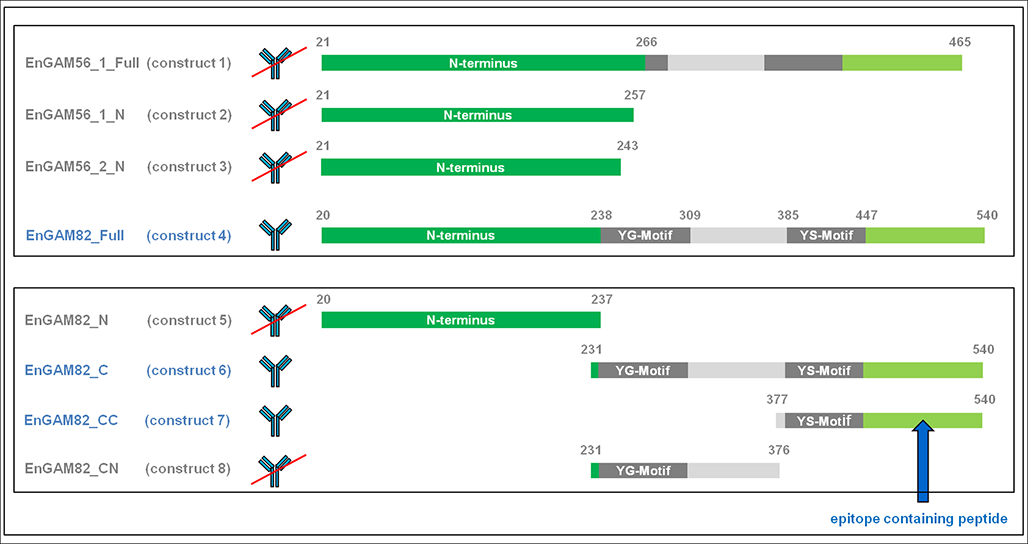

Supplement: Supplementary file 3 — Schematic illustration of heterologous expressed recombinant proteins: EnGAM56_1, EnGAM56_2, EnGAM82 and the truncated proteins of EnGAM82 (N- and C-terminus). By immunoblot of bacterial cell lysates using the mAb A1G8, the peptide EnGAM82CC (construct 7) was identified as the A1G8-epitope containing peptide (PNG 1641 kb) [file 436_2020_6765_Fig6_ESM.png]

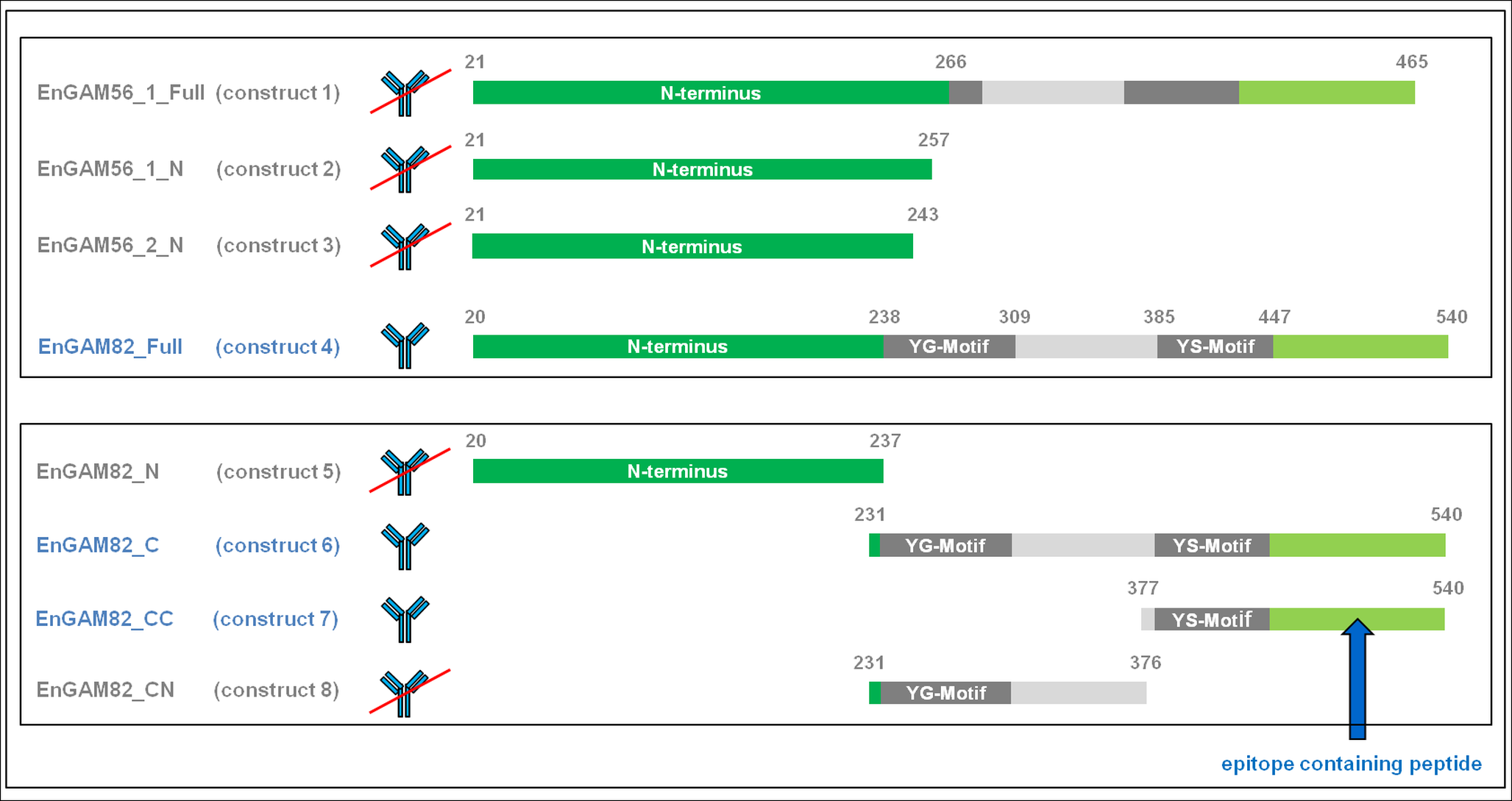

Supplement: Supplementary file 4 — High-resolution image (TIF 6565 kb) [file 436_2020_6765_MOESM2_ESM.tif]

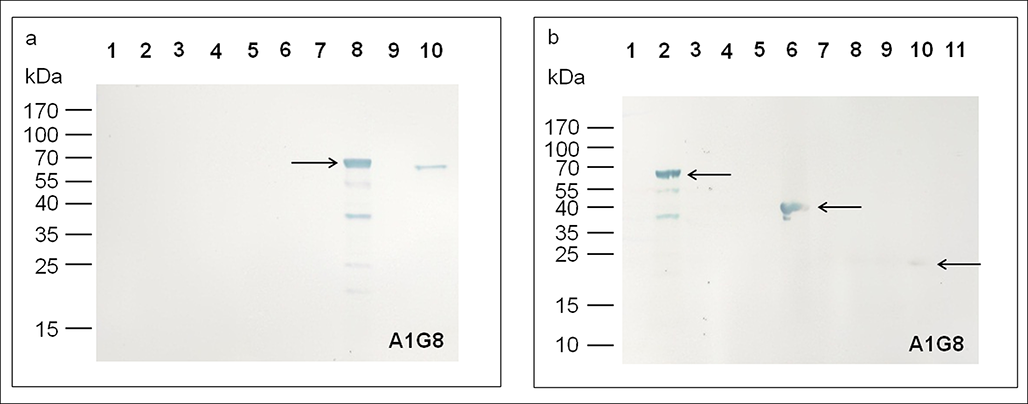

Supplement: Supplementary file 5 — FLM images of paraffin-embedded sections of E. papillata (a-c) and E. falciformis (d-f) in situ, immuno-stained with mAb A1G8, visualized with Anti-Mouse IgG-FITC and counterstained with DAPI. a Macrogametocytes of E. papillata at different developmental stages containing punctiform or circle-shaped WFBII (green), counterstained with DAPI (nuclei). b-c Circle-shaped WFBII (green) are located in the cytoplasm of the macrogametocyte (small intestine, 120 h p.i.). d-f Macrogametocytes of E. falciformis, containing circle-shaped WFBII (green), are located in crypt epithelial cells of the colon (166 h p.i.). Bar: (a) 500 μm, (c) 20 μm, (d, e) 200 μm, (f) 20 μm. FLM fluorescence light microscopy (PNG 1220 kb) [file 436_2020_6765_Fig7_ESM.png]

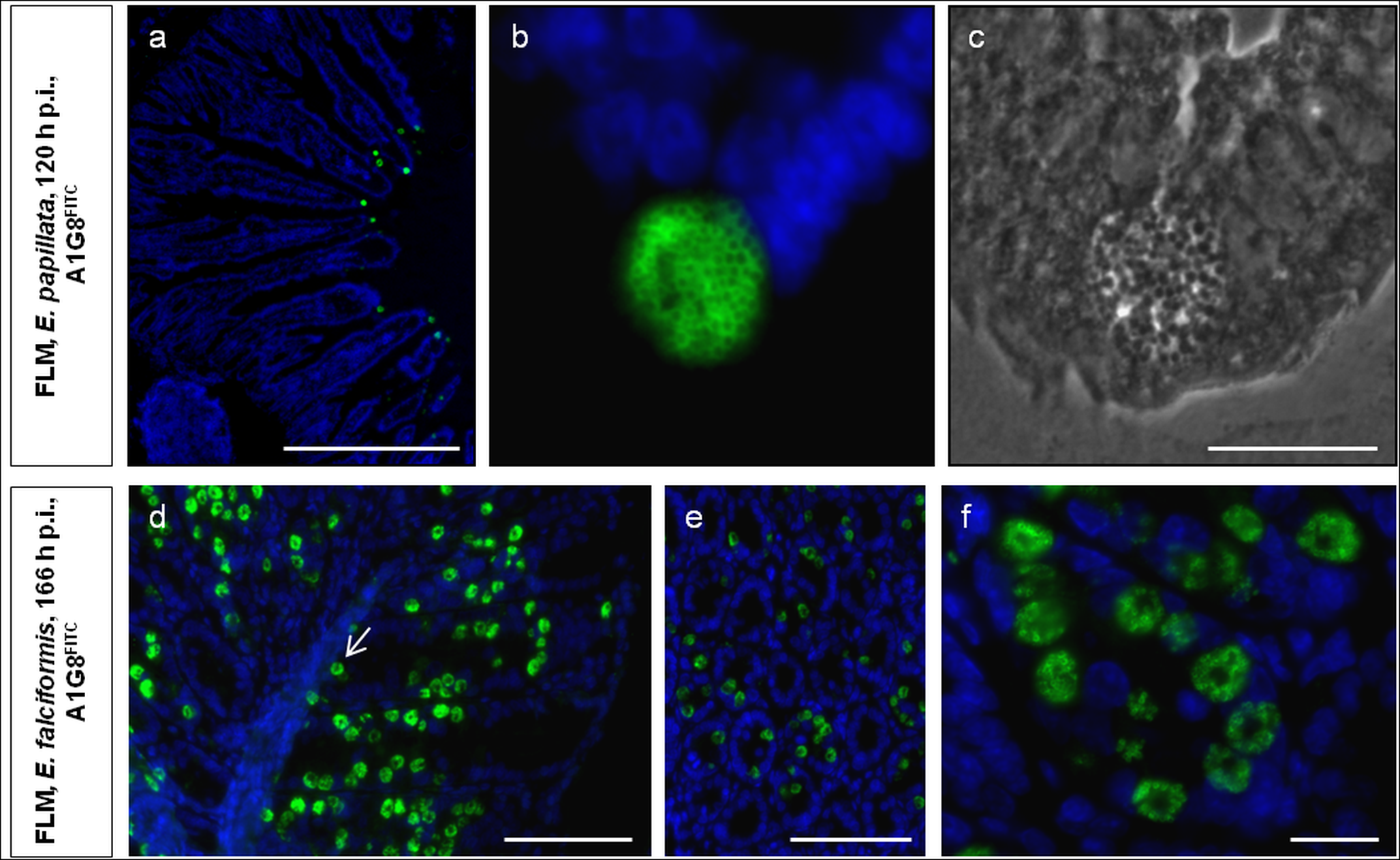

Supplement: Supplementary file 6 — High-resolution image (TIF 7613 kb) [file 436_2020_6765_MOESM3_ESM.tif]
